# Effect of MRI on preterm infants and their families: a randomised trial with nested diagnostic and economic evaluation

**DOI:** 10.1136/archdischild-2017-313102

**Published:** 2017-10-07

**Authors:** A David Edwards, Maggie E Redshaw, Nigel Kennea, Oliver Rivero-Arias, Nuria Gonzales-Cinca, Phumza Nongena, Moegamad Ederies, Shona Falconer, Andrew Chew, Omar Omar, Pollyanna Hardy, Merryl Elizabeth Harvey, Oya Eddama, Naomi Hayward, Julia Wurie, Denis Azzopardi, Mary A Rutherford, Serena Counsell

**Affiliations:** 1 Centre for the Developing Brain, School of Bioengineering and Imaging Sciences, King’s College London and Evelina London Children’s Hospital, London, UK; 2 National Perinatal Epidemiology Unit, University of Oxford, Oxford, UK; 3 Neonatal Unit, St George’s Hospital, London, UK; 4 Division of Clinical Sciences, Imperial College London, London, UK; 5 Faculty of Health, School of Midwifery, Nursing and Social Work, Birmingham City University, Birmingham, UK

**Keywords:** preterm, MRI, ultrasound, STAI, neurodevelopment

## Abstract

**Background:**

We tested the hypothesis that routine MRI would improve the care and well-being of preterm infants and their families.

**Design:**

Parallel-group randomised trial (1.1 allocation; intention-to-treat) with nested diagnostic and cost evaluations (EudraCT 2009-011602-42).

**Setting:**

Participants from 14 London hospitals, imaged at a single centre.

**Patients:**

511 infants born before 33 weeks gestation underwent both MRI and ultrasound around term. 255 were randomly allocated (siblings together) to receive only MRI results and 255 only ultrasound from a paediatrician unaware of unallocated results; one withdrew before allocation.

**Main outcome measures:**

Maternal anxiety, measured by the State-Trait Anxiety inventory (STAI) assessed in 206/214 mothers receiving MRI and 217/220 receiving ultrasound. Secondary outcomes included: prediction of neurodevelopment, health-related costs and quality of life.

**Results:**

After MRI, STAI fell from 36.81 (95% CI 35.18 to 38.44) to 32.77 (95% CI 31.54 to 34.01), 31.87 (95% CI 30.63 to 33.12) and 31.82 (95% CI 30.65 to 33.00) at 14 days, 12 and 20 months, respectively. STAI fell less after ultrasound: from 37.59 (95% CI 36.00 to 39.18) to 33.97 (95% CI 32.78 to 35.17), 33.43 (95% CI 32.22 to 34.63) and 33.63 (95% CI 32.49 to 34.77), p=0.02. There were no differences in health-related quality of life. MRI predicted moderate or severe functional motor impairment at 20 months slightly better than ultrasound (area under the receiver operator characteristic curve (CI) 0.74; 0.66 to 0.83 vs 0.64; 0.56 to 0.72, p=0.01) but cost £315 (CI £295–£336) more per infant.

**Conclusions:**

MRI increased costs and provided only modest benefits.

**Trial registration:**

ClinicalTrials.gov NCT01049594 https://clinicaltrials.gov/ct2/show/NCT01049594.

EudraCT: EudraCT: 2009-011602-42 (https://www.clinicaltrialsregister.eu/).

**What is already known on this topic?**

- Prediction of neurological outcome in preterm infants is important but difficult, and although cerebral ultrasound is widely used to assign prognosis, it is highly insensitive.
- MRI is anatomically richer and might offer greater predictive power, with better information for parents and improved selection for ongoing care.
- Parents report that brain imaging has major emotional impacts on them, but the effect of imaging information on families’ well-being has not been systematically studied.

**What this study adds?**

- Brain imaging reduces maternal anxiety and MRI slightly more than ultrasound. However, the effect is not clinically significant and does not lead to better health- related quality of life.
- MRI predicts adverse motor outcomes slightly better than ultrasound, but both methods are insensitive and neither predicts cognitive problems.
- A single MRI costs about £300 more than routine serial ultrasound.

## Introduction

Parents of preterm infants are generally aware that their infants risk neurodevelopmental impairment, and prematurity is associated with increased parental anxiety and unplanned use of healthcare after hospital discharge.^1–4^ Accurate information on an infant’s prognosis could facilitate targeting of follow-on services to affected children while reassuring families with healthy infants, reducing their anxiety and consumption of health resources.^5^

Neuroimaging is employed routinely to provide prognostic information. Cranial ultrasound is simple, inexpensive and widely used. MRI is more complex but has greater neuroanatomical definition and may have superior prognostic power.^6–8^ While there are some data on the sensitivity and specificity of these imaging modalities,^9 10^ their influence on the ongoing care and well-being of preterm infants and their families is unclear.

However, these effects are likely to be subtle and profound: parents may find the greater verisimilitude of MRI more convincing, allowing more effective reassurance of normality and acceptance of intervention for adverse outcome; conversely, incidental findings on MRI might increase parental anxiety and escalate healthcare uptake. We are unaware of studies that have systematically examined the effect of neuroimaging information on families and patient-oriented outcomes.

We therefore examined whether, compared with ultrasound, information from MRI allows more precise selection for neurodevelopmental follow-on services, decreases parental anxiety, improves health-related quality of life and reduces ongoing recourse to healthcare.

## Methods

### Study design

This parallel-group randomised controlled trial with 1:1 allocation compared the effect of prognostic information derived from either MRI or ultrasound on parental anxiety and coping, health costs and health-related quality of life. A nested diagnostic evaluation with blinded assessment compared the precision of the two investigations in selecting infants who should benefit from ongoing neurodevelopmental support and an economic evaluation estimated associated costs.

Infants were eligible if born before 33 weeks gestational age and their mother was aged over 16 years and not a hospital inpatient; they were excluded if they had major congenital malformation, prior MRI, care in a centre where preterm MRI was routine, metallic implants, parents unable to speak English or were subject to child protection proceedings.

Infants underwent both MRI and ultrasound as outpatients in a neonatal imaging centre at 38–44 weeks gestational age, then randomly allocated to receive information from one or other modality. Images were interpreted with access to the clinical history and routine clinical ultrasound reports, but not the unallocated trial imaging. An experienced physician unaware of the unallocated result discussed the allocated images and neurodevelopmental prognosis in a structured interview with parents, providing permanent examples of the images and a written summary of the prognostic information. Information from the allocated images only was then passed to the infant’s General Practitioner, Paediatrician and other medical staff, who took all decisions regarding further care.

### MRI

Standardised T1-weighted and T2-weighted MRI, including a T1-weighted dynamic scan (see table S1 in the online supplementary file 1) was performed on a 3-Tesla system (Philips Medical Systems, Best, The Netherlands) using an eight-channel phased array head coil. Pulse oximetry, temperature and heart rate were monitored throughout and ear protection was used for each infant (President Putty, Coltene Whaledent, Mahwah, New Jersey, USA; MiniMuffs, Natus Medical, San Carlos, California, USA). Chloral hydrate (25–50 mg kg^−1^) was administered to infants whose parents chose sedation for the procedure.

10.1136/archdischild-2017-313102.supp1 Supplementary file 1

MRI was interpreted using a widely accepted scheme.^11^ To predict adverse prognosis for comparison with ultrasound, we selected images with moderate or severe changes, and sensitivity analysis examined the predictive value of severe changes alone.^11^

### Cranial ultrasound

Ultrasound images were acquired using an Antares ultrasound scanner with a multifrequency transducer (Toshiba Aplio MX, Model SSA-780A) initially set at 7.0 Hz. Standardised images were acquired through the anterior fontanelle in at least six coronal and five sagittal planes.

Ultrasound images were reported using prognostic values derived by a meta-analysis published prior to the study.^10^ To predict adverse outcome for comparison, we selected images showing any feature with an individual predictive probability for cerebral palsy of greater than 25%^10^: grade 3–4 periventricular haemorrhage, periventricular leucomalacia, porencephalic cysts and/or ventricular size ≥4 mm above the 97th centile for age.

To ensure that the trial ultrasound reflected routine clinical ultrasound, we compared trial data with reports of clinical scans in hospital records.

### Outcomes

The primary outcome was maternal anxiety assessed by the state component of the State-Trait Anxiety Inventory (STAI)^12^: before the imaging visit, 14 days and 12 months after imaging and at 18–24 months corrected age, immediately prior to the child’s neurodevelopmental assessment. Mothers and fathers completed separate questionnaires. Secondary outcomes included: maternal trait anxiety, paternal state and trait anxiety, questionnaires designed to assess mothers’ coping, economic costs and health-related quality of life assessed using the preference-based instruments EQ-5D-3L and SF-12 collected at 12 and 18–24 months.^13 14^

For the nested diagnostic evaluation, children were scheduled to undergo a neurodevelopmental assessment by trained assessors blind to the allocation groups at 18–24 months corrected age. The principal reference standard was moderate or severe functional motor impairment, defined as a gross motor function classification system (GMFCS)^15^ grade 2–5. We supported this by exploring the prediction of a Bayley Scales of Infant Development III (BSID-III) motor domain score of <85.^16^ We explored the prediction of: cognitive and language abilities defined by the BSID-III cognition and language domains of <85,^16^ the parent report of children’s abilities-revised (PARCA-R)^17^ <49^17^ and the modified checklist for autism in toddlers (M-CHAT)^18^ failure of more than two critical items.

### Statistical analysis

The statistical analysis plan for the randomised trial was finalised prior to unblinding and the nested diagnostic evaluation prior to analysis, performed by independent statisticians.

The primary analysis was by repeated measures analysis of covariance using a mixed model which took account of the within-subject variability, using scores at all three postrandomisation time points and adjusting for baseline anxiety and randomisation factors. The adjusted mean group differences between baseline and each time point with 95% CIs were calculated. Missing data in the STAI were imputed if one or two questions were unanswered in each form as per the test manual; with larger numbers of missing data, the questionnaire was excluded. To avoid bias, maximise the power of the study and allow analysis by intention-to-treat, the missing-indicator method was used.^19 20^

Estimates of sample size based on an expected mean STAI total of 47, SD of 12 and correlation between follow-up measurements of 0.2 showed that 414 mothers were required to detect a mean difference of 2.5 at the 5% level with 90% power and an assessment of the rates of multiple birth and withdrawals suggested that about 510 infants would need to be randomised. The randomisation procedure employed a minimisation algorithm on a stand-alone computer that balanced site of neonatal care, gender and gestational age at birth. Siblings were allocated the same intervention.

Prespecified sensitivity analyses assessed the effects of a series of maternal characteristics and the imputation on the primary outcome. Prespecified subgroup analyses sought interactions between the treatment effect and a series of infant characteristics.

Healthcare costs were derived from the UK National Health Service perspective. Imaging-related costs were estimated using a microcosting approach in which component and unit costs were identified and valued.^21^ Data on healthcare resource use were collected at 12 and 24 months after randomisation and multiplied by unit costs (see table S2 in the online supplementary file 1) to obtain the cost per infant over the trial period. Preference-based scores for the EQ-5D-3L and SF-12 health status were derived using published algorithms.^22 23^ Missing data were imputed using a multiple imputation framework with chained equations.^24^ Mean differences between the groups and associated uncertainty in healthcare resource use, cost and health-related quality of life scores were estimated using parametric methods.^25^ More details are given in the Expanded Methods in the supplementary file 1.

To determine diagnostic precision, sensitivity, specificity, positive and negative predictive values and the area under the receiver operator characteristic curve (AUROC) were calculated and AUROC compared between modalities.

Analysis of the primary outcome and principle reference standard are presented with unadjusted p-values. Inferences from secondary hypothesis tests were made controlling the family-wise error rate using the Bonferroni method. Prognostic values are presented with 95% CI.

The study protocol was approved by the Hammersmith and Queen Charlotte’s Research Ethics Committee (09/H0707/98), and written informed consent was obtained in every case. The trial was registered prior to enrollment with the European Clinical Trials Database (EudraCT 2009-011602-42). The trial was overseen by an independent steering committee with advice from a data monitoring and ethics committee.

## Results

**Figure 1:**
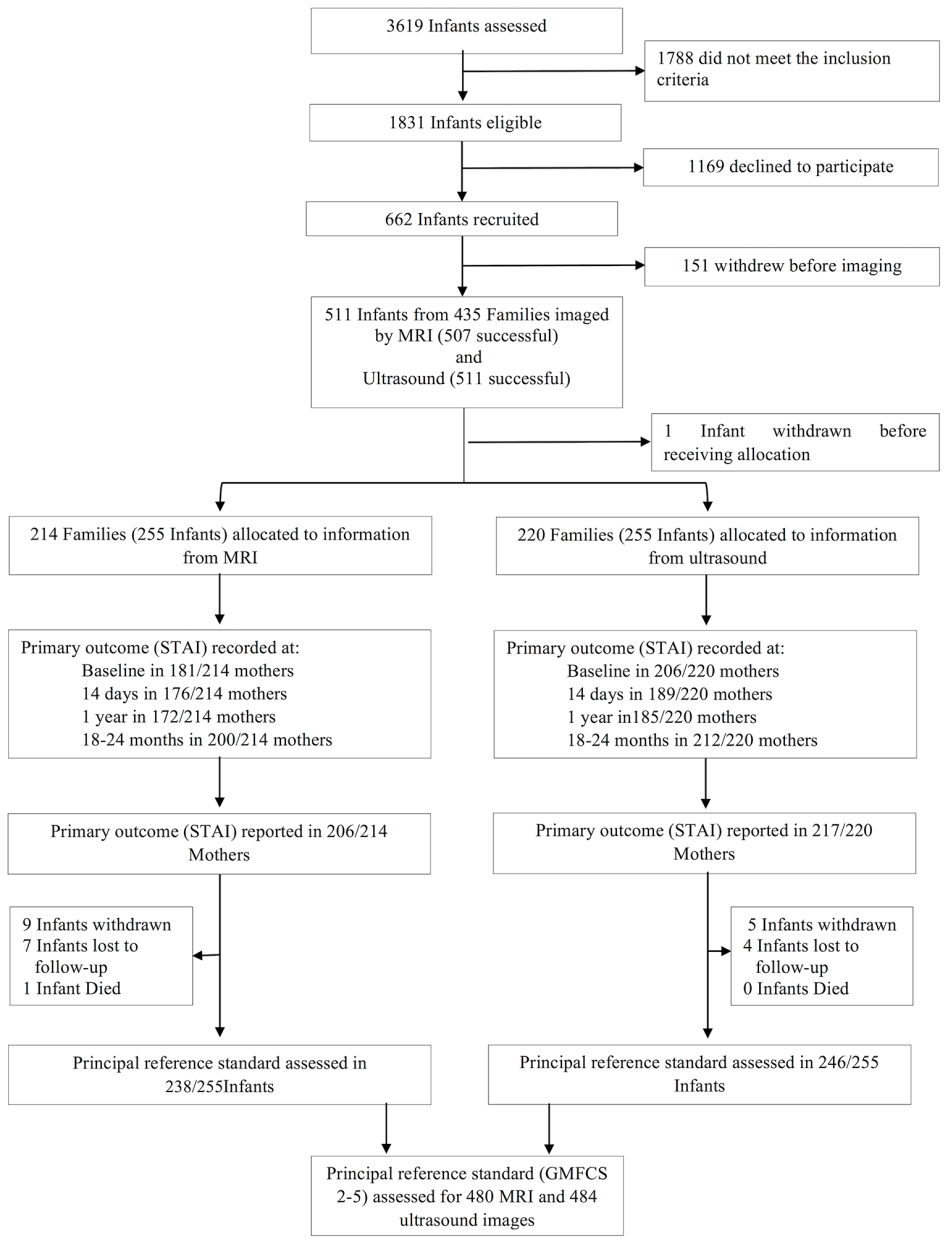
Consolidated Standards of Reporting Trials diagram for randomised study and for diagnostic evaluation. GMFCS, gross motor function classification system; STAI, State-Trait Anxiety Inventory.

Between 16 April 2010 and 31 July 2013, we screened 3619 admissions to level 1, 2 and 3 neonatal units at 14 London Hospitals and found 1831 eligible infants. Six hundred and sixty-two infants were recruited, 151 withdrew before imaging, and recruitment was closed when 511 infants in 435 families had been imaged at around term corrected age. MRI was successfully acquired in 507 infants and ultrasound in 511. One family withdrew after imaging but prior to allocation. Two hundred and fifty-five infants were randomly allocated to MRI and 255 to ultrasound. The Consolidated Standards of Reporting Trials diagram is given in figure 1, baseline characteristics of the infants and their families in table 1 and neurodevelopmental outcomes in table 2.

**Table 1:** Baseline characteristics of the infants and their families

Characteristics (families)	MRI (n=214)*	Ultrasound (n=220)*
Mother’s age at randomisation, median (IQR)	32.4 (28.8, 37.0)	32.8 (29.0, 36.6)
Maternal ethnicity, n (%)		
White	110 (52.1)	104 (47.7)
Black or Black British	38 (18.0)	55 (25.2)
Asian or Asian British	55 (26.1)	50 (22.9)
Mixed	4 (1.9)	4 (1.8)
Other ethnicity group	4 (1.9)	5 (2.3)
Missing	3	2
Mother’s age when leaving full time education, n (%)		
≤16 years	26 (12.5)	19 (9.0)
17–19 years	33 (15.9)	33 (15.6)
≥19 years	142 (68.3)	151 (71.6)
Still in full time education	7 (3.4)	8 (3.8)
Missing	6	9
Index of multiple deprivation quintiles, n(%)		
1	30 (14.0)	46 (20.9)
2	37 (17.3)	31 (14.1)
3	54 (25.2)	50 (22.7)
4	62 (29.0)	57 (25.9)
5	31 (14.5)	36 (16.4)
Mode of delivery, n (%)		
Emergency caesarean—not in labour	71 (33.5)	75 (34·4)
Emergency caesarean—in labour	49 (23.1)	43 (19·7)
Elective section—not in labour	14 (6.6)	18 (8.3)
Elective section—in labour	0 (0)	2 (0.9)
Vaginal—forceps assisted	6 (2.8)	3 (1.4)
Vaginal—spontaneous	72 (34.0)	77 (35.3)
Missing	2	2
Antenatal steroids, n (%)		
Full	179 (86.5)	183 (86.4)
Partial	30 (12.2)	28 (11.8)
None	5 (2.3)	9 (4.1)
Characteristics (infants)	MRI (n=255)†	Ultrasound (n=255)†
Sex: Male, n (%)	127 (49.8)	126 (49.4)
Gestational age at birth (weeks), median (IQR)	30 (27, 31)	30 (27, 31)
Birth weight (g), mean (SD)	1303.5 (393.0)	1306.0 (387.7)
Gestational age at scan (weeks), median (IQR)	43 (41, 44)	43 (41, 44)
Twin or triplet birth, n (%)	77 (30.2)	67 (26.3)
Surfactant treatment, n (%)	139 (54.5)	138 (54.1)
Days ventilated, median (IQR)	1 (0, 3)	1 (0, 2)
Days on CPAP, median (IQR)	7 (1, 32)	7 (2, 28)

*Number of mothers in each group (MRI and ultrasound).

†Number of infants in each group (MRI and ultrasound).

Index of multiple deprivation quintile group for a valid postcode (1, ≤8.49 (least deprived); 2, 8.5–13.79; 3, 13.8–21.35; 4, 21.36–34.17; 5: ≥34.18 (most deprived); http://tools.npeu.ox.ac.uk/imd/).

CPAP, continuous positive airway pressure.

**Table 2:** Neurodevelopmental outcomes at 18–24 months

Infant outcomes at 20–24 months	MRI (n=255)*	Ultrasound (n=255)*
Infant age at assessment, median (IQR)	20.20 (20.00–20.79)	20.20 (20.00–20.50)
Gross Motor Function Classification Scale, n (%)		
No abnormality or grade 1	221 (92.86)	229 (93.09)
Grade 2	8 (3.36)	12 (4.88)
Grade 3	3 (1.26)	2 (0.81)
Grade 4	4 (1.68)	2 (0.81)
Grade 5	2 (0.84)	1 (0.41)
Missing	17	9
Bayley-III motor composite score, mean (SD)	93.19 (14.02)	95.04 (12.62)
Missing, n	17	9
Bayley-III cognitive composite score, mean (SD)	91.16 (14.14)	92.91 (13.67)
Missing, n	17	9
Bayley-III language composite score, mean (SD)	89.23 (17.73)	91.11 (17.15)
Missing, n	17	9
PARCA-R composite, mean (SD)	45.20 (24.78)	48.02 (23.97)
Missing, n	21	10
M-CHAT failing two or more critical items, n (%)	85 (35.42)	67 (27.24)
Missing, n	15	9

*Number of infants in each group (MRI and ultrasound).

Bayley-III, Bayley Scales of Infant Development III; M-CHAT, modified checklist for autism in toddlers; PARCA-R, parent report of children’s abilities-revised.

### Primary outcome

The primary outcome was assessed in 423 mothers, 206/214 of the MRI group and 217/220 of the ultrasound group. The results are given in table 3. The STAI state score was significantly lower after imaging in the MRI group, with the largest difference between groups appearing at 1 year (overall p-value accounting for all follow-up assessments=0.02). Analysis without imputation of baseline data produced a similar conclusion (p=0.04). This difference persisted after accounting for maternal age (p=0.02), ethnicity (p=0.02), social deprivation (p=0.02), level of education (p=0.03) or prior information that the infant had an adverse outcome (p=0.05), and there was no effect of multiple births (p=0.42).

**Table 3:** State-trait anxiety inventory results (STAI)

Outcome	MRI (n=206)*	Ultrasound (n=217)*	p Value
Primary outcome			
Maternal state STAI at baseline (95% CI)	36.81 (35.18 to 38.44)	37.59 (36.00 to 39.18)	0.02
Maternal state STAI at 14 days (95% CI)	32.77 (31.54 to 34.01)	33.97 (32.78 to 35.17)	
Maternal state STAI at 12 months (95% CI)	31.87 (30.63 to 33.12)	33.43 (32.22 to 34.63)	
Maternal state STAI at 18–24 months corrected age (95% CI)	31.82 (30.65 to 33.00)	33.63 (32.49 to 34.77)	
Secondary outcomes			
Maternal trait STAI at baseline (95% CI)	37.02 (35.64 to 38.39)	38.22 (36.86 to 39.58)	0.56
Maternal trait STAI at 14 days (95% CI)	35.15 (34.03 to 36.27)	34.77 (33.70 to 35.84)	
Maternal trait STAI at 12 months (95% CI)	34.97 (33.83 to 36.10)	35.38 (34.29 to 36.47)	
Maternal trait STAI at 18–24 months corrected age (95% CI)	34.70 (33.64 to 35.77)	35.77 (34.74 to 36.81)	
Paternal state STAI	MRI (n=168)†	Ultrasound (n=176)†	
Paternal state STAI at baseline (95% CI)	34.47 (32.75 to 36.19)	35.19 (33.50 to 36.89)	0.78
Paternal state STAI at 14 days (95% CI)	32.43 (31.04 to 33.83)	32.76 (31.43 to 34.10)	
Paternal state STAI at 12 months (95% CI)	30.45 (29.01 to 31.88)	31.29 (29.93 to 32.64)	
Paternal state STAI at 18–24 months corrected age (95% CI)	31.92 (30.55 to 33.29)	31.38 (30.06 to 32.70)	
Paternal trait STAI	MRI (n=168)‡	Ultrasound (n=178)‡	
Paternal trait STAI at baseline (95% CI)	34.54 (33.12 to 35.95)	35.24 (33.78 to 36.71)	0.82
Paternal trait STAI at 14 days (95% CI)	33.15 (31.93 to 34.36)	33.15 (32.00 to 34.31)	
Paternal trait STAI at 12 months (95% CI)	32.10 (30.85 to 33.35)	33.13 (31.95 to 34.31)	
Paternal trait STAI at 18–24 months corrected age (95% CI)	33.43 (32.23 to 34.63)	32.86 (31.72 to 34.01)	

*Number of mothers with primary and secondary outcome data (STAI) in each group (MRI and ultrasound) at baseline, 14 days, 12 months and 18–24 months corrected age,

†Number of fathers with secondary outcome data (state STAI) in each group (MRI and ultrasound) at baseline, 14 days, 12 months and 18–24 months corrected age.

‡Number of fathers with secondary outcome data (trait STAI) in each group (MRI and ultrasound) at baseline, 14 days, 12 months and 18–24 months corrected age.

Anxiety scores for mothers whose infants did not develop moderate or severe functional motor impairment were lower for the MRI group, while for those whose infants did were higher (p-value for interaction=0.01). The difference between the groups was not influenced by gestational age at birth (p=0.43), need for mechanical ventilation (p=0.82) or whether the allocated imaging predicted adverse outcome (0.87).

### Secondary outcomes

There was no significant difference in maternal STAI (p=0.55), paternal state (0.77) or trait anxiety (p=0.81).

There were no significant differences in health-related quality of life (see table S3 in the online supplementary file 1). No significant differences were observed in any category of healthcare resource use (see table S4 in the online supplementary file 1). Mean costs of delivering a single MRI scan and routine clinical ultrasound were £773 and £458, respectively, a significant mean cost difference (95% CI) of £315 (£295–£336) per infant (see table S5 in the online supplementary file 1). The total mean cost per infant over the 24 months follow-up was £16 231 and £10 916 for the MRI and ultrasound groups, respectively, a non-significant mean cost difference (95% CI) of £5315 (−£188 to £10 819). To describe maternal confidence in caring for their infants further, we report the results of relevant questionnaires in table S6 in the online supplementary file 1. These show most mothers coping well in both groups.

### Diagnostic precision

Twenty-two infants had ultrasound scans and 72 had MRI scans predicting adverse prognosis. GMFCS and BSID-III were assessed in 484 infants at median 20.18 (IQR 20.00–20.66) months corrected age. Sensitivity, specificity, positive and negative predictive values and AUROC could be calculated for 480 MRI and 484 ultrasound images; results are given in table 4. Trial and routine clinical ultrasound were compared in the 420 infants where clinical reports were available and showed no difference: trial AUROC 0.66 (0.58–0.74), routine 0.68 (0.59–0.77293), (p=0.45).

**Table 4:** Predictive values of MRI and ultrasound

MRI (n=480)*								Ultrasound (n=484)^*^			
Outcome	Sensitivity (95% CI)	Specificity (95% CI)	PPV (95% CI)	NPV (95% CI)	AUROC (95% CI)	Sensitivity (95% CI)	Specificity (95% CI)	PPV (95% CI)	NPV (95% CI)	AUROC (95% CI)	p Value
Principle reference standard (moderate or severe functional impairment)											
GMFCS grade 2–5	60.6 (42.1 to 77.1)	88.4 (85.0 to 91.2)	27.8 (17.9 to 39.6)	96.8 (94.6 to 98.3)	0.74 (0.66 to 0.83)	29.4 (15.1 to 47.5)	97.6 (95.7 to 98.8)	47.6 (25.7 to 70.2)	94.8 (92.4 to 96.7)	0.64 (0.56 to 0.72)	0.01
Secondary reference standards											
Bayley-III motor domain score<85	38.9 (27.6 to 51.1)	89.2 (85.8 to 92.1)	38.9 (27·6 to 51.1)	89.2 (85.8 to 92.1)	0.64 (0.58 to 0.70)	16.4 (8.8 to 27.0)	97.8 (95.9 to 99.0)	57.1 (34.0 to 78.2)	86.8 (83.4 to 89.8)	0.57 (0.53 to 0.62)	0.008
Bayley-III cognitive domain score<85	27.9 (19.8 to 37.2)	88·9 (85.2 to 91.9)	43.1 (31.4 to 55.3)	80.4 (76.2 to 84.1)	0.58 (0.54 to 0.63)	12.5 (7.0 to 20.1)	98.1 (96.2 to 99.2)	66.7 (43.0 to 85.4)	78.8 (74.8 to 82.5)	0.55 (0.52 to 0.59)	0.13
Bayley-III language domain score<85	19.9 (14.3 to 26.6)	87.8 (83.6 to 91.3)	48.6 (36.7 to 60.7)	65.4 (60.6 to 70.1)	0.54 (0.50 to 0.57)	6.2 (3.1 to 10.8)	96.7 (94.1 to 98.4)	52.4 (29.8 to 74.3)	63.9 (59.4 to 68.3)	0.51 (0.49 to 0.54)	0.14
PARCA-R index score<49	18.7 (14.3 to 23.8)	91·9 (87.1 to 95.3)	76.5 (64.6 to 85.9)	44.5 (39.6 to 49.4)	0.55 (0.52 to 0.58)	5.3 (3.0 to 8.7)	97.0 (93.5 to 98.9)	71.4 (47.8 to 88.7)	41.9 (37.4 to 46.6)	0.51 (0.49 to 0.53)	0.003
M-CHAT failure of more than two critical items	23.8 (17.3 to 31.4)	89.1 (85.3 to 92.3)	50.0 (38.0 to 62.0)	72.0 (67.3 to 76.2)	0.56 (0.53 to 0.60)	6.6 (3.2 to 11.8)	96.7 (94.2 to 98.3)	47.6 (25.7 to 70.2)	69.5 (65.1 to 73.6)	0.52 (0.49 to 0.54)	0.006

*Number of MRI or ultrasound images and infants with principal reference standard assessed (missing, n: PARCA-R: MRI (21), ultrasound (10)); M-CHAT: MRI (15); ultrasound (9).

AUROC, area under the receiver operator characteristics curve; Bayley-III, Bayley Scales of Infant Development III; GMFCS, gross motor function classification system; M-CHAT, modified checklist for autism in toddlers; NPV, negative predictive value; PPV, positive predictive value; PARCA-R, parent report of children’s abilities-revised.

Evaluating the principal reference standard, MRI was more predictive of moderate or severe functional impairment than ultrasound (AUROC: MRI (0.74 (0.66–0.83); ultrasound (0.64 (0.56–0.72), (p=0.01)) and more predictive of outcome in the BSID-III motor domain (AUROC: MRI 0.64 (0.58 to 0.70); ultrasound 0.57 (0.53 to 0.61), (p=0.008). Severe changes alone on MRI produced lower AUROC.

Prediction of PARCA-R was assessed in 475 infants and M-CHAT in 482. AUROC for all neurocognitive and language domain tests were less than 0.6, although better by MRI for M-CHAT (p=0.006) and PARCA-R (p=0.003), and sensitivity was low.

## Discussion

Preterm birth has long-lasting effects on individuals and families, and increased maternal anxiety adversely influences child development.^3 4 26^ This study describes the wider effects of information on the lives of preterm infants and their families.

Mothers’ anxiety was reduced in both groups after they received information from neuroimaging, slightly more after MRI. This effect was not limited to a particular group of mothers or infants, was unaffected by the severity of the infants’ medical problems and was not a reflection of long-term anxiety traits; it was nuanced with MRI increasing anxiety in mothers whose children had emerging impairments. Though statistically significant, the effect was of little clinical significance. The STAI was persistently less than 40, and thus regarded as normal, and imaging information did not alter health-related quality of life or decrease the need for infant healthcare and had no effects on fathers. However, the results show that imaging information does not increase family anxiety, and the statistically significant difference between the groups is evidence that the study was correctly powered and a type 2 error unlikely.

Prediction of adverse outcome is an important aspect of neonatal care.^27^ Our principal reference standard for diagnostic assessment was moderate or severe functional motor impairment (GMFCS 2–5),^28^ which is frequently used in preterm infants^29^ and reduces the variability inherent in distinguishing mild impairment from normal at this age. It has been suggested that GMFCS is less stable before age 2,^30^ but comparable predictions were made of suspected motor impairment defined by the BSID-III, an established test which correlates with, although probably underestimates, long-term motor deficits.^31^ MRI was a better, but still imperfect predictor, selecting 15% of survivors of whom one-quarter had moderate or severe functional motor deficits while missing approximately one-third of children with neuromotor problems.

As well as motor function, neurocognitive abilities are important in determining long-term outcomes, particularly in children of economically disadvantaged families,^32–35^ and neurocognitive testing around 2 years stably predicts later ability.^36 37^ Neither MRI nor ultrasound provided precise prediction of cognitive or language function, or an increased risk of autism.

These results appear to be robust. The sample comprises over 25% of the eligible patients from a diverse range of neonatal units, more than in the largest systematic review,^9^ and neurodevelopmental outcomes were typical of the preterm population.^38 39^ MRI was acquired in 99% of infants and interpreted using a widely available scheme.^9 11^ We did not study volumetric or diffusion MRI: despite predictive power in group studies^6 40 41^ they often involve specific computational methods. Trial ultrasound had similar prognostic value to routine clinical examinations, and the values used to inform parents were broadly supported by this larger cohort.^11^ Results were unchanged by the sensitivity analyses.

MRI thus did no harm, but provided only modest improvements in patient and family outcomes, while increasing the cost of care by around £315 per patient. These data do not suggest that all preterm infants should be offered MRI.
