# Supplementary material for: Effect of MRI on preterm infants and their families: a randomised trial with nested diagnostic and economic evaluation
Source: Arch Dis Child Fetal Neonatal Ed. 2017 Oct 7;103(1):F15–21. doi: 10.1136/archdischild-2017-313102 (PMC5750369; doi:10.1136/archdischild-2017-313102)
Supplement: Supplementary file 1 [file fetalneonatal-2017-313102supp001.docx]

Supplementary Appendix

**LIST OF CONTENTS**

1 Expanded methods: healthcare costs and health-related quality of life analysis 4

1.1 Intervention costs 4

1.2 Healthcare resource use 5

1.3 Unit cost data collection 5

1.4 Cost-analysis 6

1.5 Health-related quality of life (HRQoL) 6

1.6 Statistical analysis 7

2 CONSORT checklists and supplementary information 8

2.1 CONSORT checklist 8

2.2 CONSORT Extension for Abstracts 11

2.3 Methods 12

2.3.1 Changes to the methods after trial commencement (3b) 12

2.3.2 Sample size and power (6b/7a) 13

2.3.3 Interim analysis and stopping guidelines (unmasking of randomized intervention) (7b) 13

2.3.4 Randomization (8a) 14

2.3.5 Type of randomisation (8b/9/10) 14

2.4 Minimization 14

2.4.1 Blinding (11a) 14

2.4.2 Methods for additional analysis (12b) 15

2.5 Results 15

2.5.1 Recruitment period (14a) 15

2.5.2 Harms (19) 16

2.6 Discussion 16

2.6.1 Trial limitations (20) 16

2.7 Other information 16

2.7.1 Trial protocol can be accessed (24) 16

2.7.2 Funding (25) 16

3 STARD 2015 checklist and supplementary information 18

3.1 STARD checklist 18

3.2 Methods 20

3.2.1 Participants (8/9) 20

3.2.2 Sample size (18) 20

4 CHEERS checklist and supplementary information 21

4.1 CHEERS checklist 21

5 Governance Committees 25

5.1 Trial Steering Committee 25

5.2 Data monitoring and Ethics Committee 25

6 Reference list 26

7 SUPPLEMENTARY TABLES 27

**Index of supplementary tables**

[Table S1: Parameters for MR imaging 17](#_Toc442960479)

[Table S2: Summary of categories of health care resource use items and unit costs used in the cost-analysis (expressed in 2013/2014 UK prices) 18](#_Toc442960480)

[Table S3: Maternal health-related quality of life using EQ-5D-3L and SF-6D scores at 12 and 20 months follow-up using multiple imputation analysis 20](#_Toc442960481)

[Table S4: Breakdown of infant healthcare resource use consumption over the past 24 months since leaving neonatal care, complete cases only 21](#_Toc442960482)

[Table S5: Cost-analysis over the past 20 months since leaving neonatal care expressed in 2013/2014 UK prices using multiple imputation analysis 23](#_Toc442960483)

[Table S6: Maternal confidence and perceptions of singleton infants and their care (Questionnaire data from 4 time points) 25](#_Toc442960484)

# Expanded methods: healthcare costs and health-related quality of life analysis

## Intervention costs

A micro-costing approach, where each component of resource use and unit costs related to the interventions was identified and valued, was implemented to estimate the intervention costs for MRI and ultrasound ^1^. To identify the key components of the interventions, the health economics team consulted with a consultant neonatologist from St Thomas’ Hospital, a second consultant neonatologist from Hammersmith Hospital, and a clinical technologist from Hammersmith Hospital in London. Equipment and staff were identified in the interviews as the main cost drivers of delivering MRI and ultrasound information in practice. Additional elements were also identified and categorized in a miscellaneous group.

The equivalent annual cost of equipment (MRI or ultrasound) was calculated using standard methods that incorporate useful life and resale value of the equipment at an annual rate of 5% ^1^. The cost of construction and building work to allocate a new MRI suite in a large neonatal service unit was included in the purchase price of the equipment in the base case calculation. The cost of equipment per preterm baby was estimated by dividing the annual cost of equipment attributable to preterm babies by the number of preterm babies requiring MRI or ultrasound. For MRI, it was assumed that a proportion of the annual costs of the equipment was attributable to preterm babies. This reflected that MR equipment in a large neonatal service unit has spare capacity for other children. To determine such proportion information about the annual number of preterm babies requiring MRI, average scan time (in hours) and the number of annual usage hours of MRI equipment was needed. The annual number of preterm babies requiring MR images in a large neonatal service unit was obtained from the 2013 Child Services Annual Report from Imperial College Healthcare NHS Trust ^2^. The average scan time was elicited from an expert consultant neonatologist from St Thomas’ Hospital and estimated to be 1.5 hours per MRI. It was assumed that MRI equipment was used 40 hours a week and the finance department at St Thomas’ Hospital confirmed that maintenance was necessary for two months a year. This yielded a total number of usage hours per year of 1,600 (10 months at 40 hours a week). For ultrasound, it was also assumed that a proportion of the total annual cost of the equipment was attributable to preterm babies. In this case, such proportion was estimated as the ratio of the annual number of preterm babies to the annual admissions to neonatal services. This reflected that ultrasound equipment was normally shared across all admissions to the neonatal unit. In this case the information about the annual number of preterm babies and annual admissions to neonatal services were also extracted from the 2013 Child Services Annual Report ^2^.

Staff were categorized by the health care professionals who delivered the intervention on site, a paediatric crash team and hospitality services. Miscellaneous costs for MRI included drug costs and cost of an empty neonatal room whereas for ultrasound included gel sachet and printing paper. The total cost of delivering MRI or ultrasound was estimated per preterm baby by adding the equipment, staff and miscellaneous costs.

## Healthcare resource use

Detailed information about child usage of community professionals, secondary care and prescribed drugs (other than antibiotics) since leaving neonatal care was collected at 12 and 20 months follow-up using postal questionnaires.

## Unit cost data collection

Equipment and miscellaneous unit costs information related to the interventions was obtained primarily from the finance department at St Thomas’ Hospital and a clinical technologist at Hammersmith Hospital in London. Unit costs for health care professionals and secondary care were extracted from national sources including the Personal Social Services Research Unit (PSSRU) ^3^, and the 2013 NHS Reference costs ^4^. All unit costs were expressed in 2013-2014 pounds sterling inflated to this base using the Hospital and Community Health Service Inflation Index where appropriate and are presented in Supplementary Table 2. Costs incurred between 12 and 24 months follow-up were discounted at an annual rate of 3.5% as currently recommended ^5^.

## Cost-analysis

Volumes of categories of resource use were multiplied by the corresponding unit cost to estimate the cost per preterm baby in a particular category of health care costs. Babies of mothers randomized to the MRI arm of the study were assigned the cost of receiving one MRI. As advised by the consultant neonatologists from Hammersmith Hospital, for babies of mothers randomized to the ultrasound group, we costed 8 ultrasounds for preterm babies with gestational age less than or equal 31 weeks and 2 ultrasounds for preterm babies greater than 31 weeks. When collecting data about use of medicine and drugs, we collected information about the type of drug and the length of course but not about specific doses. As no differences in the number of prescriptions and days using medication was observed between the groups at 12 and 20 months follow-up, the overall total cost per preterm baby at 24 months follow-up excluded the cost of prescribed medications. We asked mothers whether their child had had an operation at 12 and 24 months follow-up. For that category, reliable information about length of stay but not on the type of operation was available and the former was used in the final cost analysis. The mean cost per preterm baby of a particular healthcare category was estimated by averaging across children in each trial arm. Categories of resource use and associated costs are presented separately as recommended by recent guidance ^6^.

## Health-related quality of life (HRQoL)

The preference-based HRQoL instruments, EQ-5D-3L and SF-12, were used to collect maternal quality of life information at 12 and 20 months follow-up. No preference-based HRQoL was included in the battery of instruments collecting information at baseline. The EQ-5D-3L is a multi-attribute generic instrument for measuring HRQoL and widely used in economic evaluation of health care technologies ^7^. Its descriptive system covers 5 dimensions (mobility, self-care, usual activity, pain/discomfort, and anxiety/depression), each of which has 3 levels (no problem, some problems, and extreme problems). The 243 health states of the EQ-5D-3L can be converted into preference-based utility values using a value set obtained from a representative sample of the British general population ^8^. The 12-item Short-Form Health Survey (SF-12) is a generic HRQoL instrument derived from the 36-item Short-Form (SF-36) ^9^, which was designed to measure general health functioning. The SF-12 items measure physical or emotional limitations, physical functioning, pain, general health, vitality, social functioning, and mental health problems. There are two versions available and in this study the SF-12v2 was administered. We used the SF-6D algorithm to convert health states from the SF-12 instrument into preference-based utilities ^10^

## Statistical analysis

Recent evidence suggests that both parametric and non-parametric methods accurately estimate the true standard errors of means even when data are highly skewed and moderate to large (n>50) sample sizes for continuous variables ^11^. Therefore, mean differences between intervention arms and associated uncertainty in healthcare resource use, cost and HRQoL scores were estimated using parametric methods. A 95% significance level was used to compare main categories of healthcare resource use, costs and HRQoL between treatment arms. Missing EQ-5D-3L index and SF-6D utilities at 12 and 24 months follow-up and missing costs over the 24 month time horizon were imputed using a multiple imputation framework with chained equation ^12^. We used recent guidance on handling missing data in cost-effectiveness analysis to inform such analysis ^13^. We constructed an imputation model that included baseline information with complete data, EQ-5D-3L and SF-6D utility variables, and all individual categories of cost variables. Baseline information included maternal age, sex of infant, gestational age (in weeks), baby’s birth weight, and days in neonatal care. Maternal age was missing for one mother and was imputed using conditional mean imputation before including it in the multiple imputation model. We used prediction mean matching, estimated 50 different imputations, and the imputation model was implemented separately by trial allocation. Mean estimates (and associated standard errors) and mean differences (and associated 95% confidence intervals) were combined between imputed datasets using Rubin’s rule ^14^. All analyses were carried out in StataMP version 13 [StataCorp. 2013. Stata Statistical Software: Release 13. College Station, TX: StataCorp LP.]

# CONSORT checklists and supplementary information

## CONSORT checklist


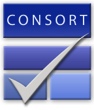
CONSORT 2010 checklist of information to include when reporting a randomised trial*

| Section/Topic | Item No | Checklist item | Reported on |
| --- | --- | --- | --- |
| Title and abstract | | | |
|  | 1a | Identification as a randomised trial in the title | Paper |
|  | 1b | Structured summary of trial design, methods, results, and conclusions (for specific guidance see CONSORT for abstracts) | Paper |
| Introduction | | | |
| Background and objectives | 2a | Scientific background and explanation of rationale | Paper |
|  | 2b | Specific objectives or hypotheses | Paper |
| Methods | | | |
| Trial design | 3a | Description of trial design (such as parallel, factorial) including allocation ratio | Paper |
|  | 3b | Important changes to methods after trial commencement (such as eligibility criteria), with reasons | Appendix |
| Participants | 4a | Eligibility criteria for participants | Paper |
|  | 4b | Settings and locations where the data were collected | Paper |
| Interventions | 5 | The interventions for each group with sufficient details to allow replication, including how and when they were actually administered | Paper |
| Outcomes | 6a | Completely defined pre-specified primary and secondary outcome measures, including how and when they were assessed | Paper |
|  | 6b | Any changes to trial outcomes after the trial commenced, with reasons | Appendix |
| Sample size | 7a | How sample size was determined | Paper and Appendix |
|  | 7b | When applicable, explanation of any interim analyses and stopping guidelines | Appendix |
| Randomisation: |  |  |  |
| Sequence generation | 8a | Method used to generate the random allocation sequence | Paper and Appendix |
|  | 8b | Type of randomisation; details of any restriction (such as blocking and block size) | Paper and Appendix |
| Allocation concealment mechanism | 9 | Mechanism used to implement the random allocation sequence (such as sequentially numbered containers), describing any steps taken to conceal the sequence until interventions were assigned | Paper and Appendix |
| Implementation | 10 | Who generated the random allocation sequence, who enrolled participants, and who assigned participants to interventions | Paper and Appendix |
| Blinding | 11a | If done, who was blinded after assignment to interventions (for example, participants, care providers, those assessing outcomes) and how | Paper and Appendix |
|  | 11b | If relevant, description of the similarity of interventions | Not applicable |
| Statistical methods | 12a | Statistical methods used to compare groups for primary and secondary outcomes | Paper |
|  | 12b | Methods for additional analyses, such as subgroup analyses and adjusted analyses | Paper and Appendix |
| Results | | | |
| Participant flow (a diagram is strongly recommended) | 13a | For each group, the numbers of participants who were randomly assigned, received intended treatment, and were analysed for the primary outcome | Paper |
|  | 13b | For each group, losses and exclusions after randomisation, together with reasons | Paper |
| Recruitment | 14a | Dates defining the periods of recruitment and follow-up | Appendix |
|  | 14b | Why the trial ended or was stopped | Not applicable |
| Baseline data | 15 | A table showing baseline demographic and clinical characteristics for each group | Paper |
| Numbers analysed | 16 | For each group, number of participants (denominator) included in each analysis and whether the analysis was by original assigned groups | Paper |
| Outcomes and estimation | 17a | For each primary and secondary outcome, results for each group, and the estimated effect size and its precision (such as 95% confidence interval) | Paper |
|  | 17b | For binary outcomes, presentation of both absolute and relative effect sizes is recommended | Not applicable |
| Ancillary analyses | 18 | Results of any other analyses performed, including subgroup analyses and adjusted analyses, distinguishing pre-specified from exploratory | Paper |
| Harms | 19 | All important harms or unintended effects in each group (for specific guidance see CONSORT for harms) | Appendix |
| Discussion | | | |
| Limitations | 20 | Trial limitations, addressing sources of potential bias, imprecision, and, if relevant, multiplicity of analyses | Appendix |
| Generalisability | 21 | Generalisability (external validity, applicability) of the trial findings | Paper |
| Interpretation | 22 | Interpretation consistent with results, balancing benefits and harms, and considering other relevant evidence | Paper |
| Other information | | |  |
| Registration | 23 | Registration number and name of trial registry | Paper |
| Protocol | 24 | Where the full trial protocol can be accessed, if available | Paper and Appendix |
| Funding | 25 | Sources of funding and other support (such as supply of drugs), role of funders | Paper and Appendix |

## CONSORT Extension for Abstracts

| **Item** | **Description** | **Reported on** |
| --- | --- | --- |
| Title | Identification of the study as randomized | Yes |
| Authors * | Contact details for the corresponding author | Not applicable |
| Trial design | Description of the trial design (e.g. parallel, cluster, non-inferiority) | Yes |
| Methods |  |  |
| Participants | Eligibility criteria for participants and the settings where the data were collected | Yes |
| Interventions | Interventions intended for each group | Yes |
| Objective | Specific objective or hypothesis | Yes |
| Outcome | Clearly defined primary outcome for this report | Yes |
| Randomization | How participants were allocated to interventions | Yes |
| Blinding (masking) | Whether or not participants, care givers, and those assessing the outcomes were blinded to group assignment | Yes |
| Results |  |  |
| Numbers randomized | Number of participants randomized to each group | Yes |
| Recruitment | Trial status | Yes |
| Numbers analysed | Number of participants analysed in each group | Yes |
| Outcome | For the primary outcome, a result for each group and the estimated effect size and its precision | Yes |
| Harms | Important adverse events or side effects | No |
| Conclusions | General interpretation of the results | Yes |
| Trial registration | Registration number and name of trial register | Yes |
| Funding | Source of funding | No |

## Methods

### Changes to the methods after trial commencement (3b)

The programme is that proposed in the research plan, with the exception that the sample size needed to detect a significant primary outcome in the randomized trial was re-assessed after 234 patients were recruited. The high level compliance with outcome testing together with low within-subject variance allowed us to consider repeated measures ANCOVA for the primary analysis, with a concomitant reduction in sample size. The trial sample size was therefore re-planned.

### Sample size and power (6b/7a)

The STAI measure of state anxiety was the primary outcome. The initial power calculations were based on a trial with about 2n=820 in each group for a range of standard deviations assuming analysis using a two-sided, two-sample t-test with a significance level of 5% (alpha=0.05). The trial was large enough to detect a difference in mean STAI state anxiety score of 2.5 with a power of 90%, based on a standard deviation of 12.8.

Review based on the data available when 234 subjects had been studied showed that there were unexpectedly high rates of completion and within-subject correlation in the 4 STAI questionnaires, and that it would be appropriate to use repeated measures ANCOVA, taking account of the measurements at baseline and three follow up time points. This analysis of the preliminary data showed the within-subject correlation in STAI was between 0.2 and 0.5 and the standard deviation of the measurements is 12. This approach led to a significant increase in power, and the study was reset to an estimated sample size of 2n = 414. Based on this analysis, and after consideration of its effect on other projects and the rates of multiple birth and withdrawals, the estimated sample size was re-set to 510 subjects.

### Interim analysis and stopping guidelines (unmasking of randomized intervention) (7b)

There was no interim analysis and no stopping rules were defined. This was not a masked intervention, although follow up data were obtained and assessed without information being given on allocation.

### Randomization (8a)

Families were informed about the trial as inpatients. Written consent to participate was obtained during the imaging visit, and, using a stand-alone computer program, randomization was undertaken after imaging was completed, and the patients then allocated to the intervention groups. Randomization after imaging was to prevent expectation of imaging information leading to bias in attendance rates. Parents who decided at this stage not to be involved in the trial were to be recorded as leaving the trial and were not given information from either imaging modality; in fact the one withdrawal from the study was a mother who felt unable to be given any information, so this element of the protocol was not implemented.

### Type of randomisation (8b/9/10)

Infants were randomized after imaging and definition of prognosis from the images. To make sure the groups could be compared properly the choice of which scan results were revealed was made at random using a computer program, so that there was an equal chance of receiving either the MR or the ultrasound results. Randomization used a minimization algorithm to balance on the following factors: 14 different hospitals, sex (male/female), and individual week of gestation at birth (10 groups: 23^+0^–23^+6^, 24^+0^–24^+6^… 32^+0^–32^+6^).

Multiple births were randomized with siblings allocated to the same intervention, but as the experience of a mother with more than one infant in the study may be very different, a sensitivity analysis adjusting for the effect of multiple birth was specified.

## Minimization

Gestational age, center and gender were minimized to ensure balance in each group.

### Blinding (11a)

In this trial the parents knew which modality the information they were receiving was derived from. This was essential because an important part of the information passed to the parents was to show the them the relevant brain images and the gestalt effect of seeing high resolution MR or low resolution CUS was expected to have an effect on their response to the information they were given. However, the trial was pursued and assessed blind to allocation; information-givers, families and healthcare professionals looking after the child were all blinded to the scan results contained in the non-disclosed imaging modality. After the conclusion of the trial, parents were given this information as well. Neurodevelopmental assessors were blind to allocation, and the statistical analysis plan was finalized prior to unmasking of allocation.

### Methods for additional analysis (12b)

Significance levels and adjustment of p-values for multiplicity

This is addressed in the main paper

Subgroup analysis

These are specified in the main paper.

Analysis of interactions

These are specified in the main paper.

Sensitivity and exploratory analysis

These are specified in the main paper.

## Results

### Recruitment period (14a)

The study opened on 16th April 2010. The first patient was recruited on 19^th^ May 2010. The last patient was imaged and randomized on 31^st^ July 2013. The neurodevelopmental assessments were carried out from 16^th^ April 2012 to 24^th^ April 2015. The trial ended when the re-planned recruitment was completed.

### Harms (19)

No serious adverse effects were noted.

## Discussion

### Trial limitations (20)

We were not able to take a fully geographic sample as not all hospitals were able to participate, however the demographic data on the sample give confidence that this is unlikely to have made a significant imbalance. A second limitation was the exclusion of non-English speakers, which was necessary as the use of interpreters would have made it impossible to know whether the structured interview was carried out appropriately; our results thus formally only apply to English speaking families, although there is no strong reason to believe that they are not more widely applicable.

## Other information

### Trial protocol can be accessed (24)

The trial protocol is available at:

URL: <https://clinicaltrials.gov/show/NCT01049594>

### Funding (25)

Imperial College Healthcare Trust (ICHT) was awarded funding by the National Institute for Health and Research (NIHR) Programme Grants for Applied Research (PGfAR) scheme for the project “Evaluation of MR imaging to predict neurodevelopmental impairment in preterm infants” (ePrime) with reference number RP-PG-0707-10154. ICHT entered into a contract dated 22/01/2009 with the Secretary of State for Health to undertake the project. The NIHR awarded the ePrime grant in March 2009. The Chief Investigator was Professor David Edwards.

Subcontracts between ICHT and the three collaborating institutions: Imperial College London; the National Perinatal Epidemiology Unit (NPEU); University of Oxford and St George’s NHS Foundation Trust were established.

#

# STARD 2015 checklist and supplementary information

## STARD checklist

|  | **Section & Topic** | **No** | **Item** | **Reported on** |
| --- | --- | --- | --- | --- |
|  |  |  |  |  |
|  | **TITLE OR ABSTRACT** |  |  |  |
|  |  | **1** | Identification as a study of diagnostic accuracy using at least one measure of accuracy  (such as sensitivity, specificity, predictive values, or AUC) | Paper |
|  | **ABSTRACT** |  |  |  |
|  |  | **2** | Structured summary of study design, methods, results, and conclusions  (for specific guidance, see STARD for Abstracts) | Paper |
|  | **INTRODUCTION** |  |  |  |
|  |  | **3** | Scientific and clinical background, including the intended use and clinical role of the index test | Paper |
|  |  | **4** | Study objectives and hypotheses | Paper |
|  | **METHODS** |  |  |  |
|  | *Study design* | **5** | Whether data collection was planned before the index test and reference standard  were performed (prospective study) or after (retrospective study) | Paper |
|  | *Participants* | **6** | Eligibility criteria | Paper |
|  |  | **7** | On what basis potentially eligible participants were identified  (such as symptoms, results from previous tests, inclusion in registry) | Paper |
|  |  | **8** | Where and when potentially eligible participants were identified (setting, location and dates) | Paper and Appendix |
|  |  | **9** | Whether participants formed a consecutive, random or convenience series | Paper and Appendix |
|  | *Test methods* | **10a** | Index test, in sufficient detail to allow replication | Paper |
|  |  | **10b** | Reference standard, in sufficient detail to allow replication | Paper |
|  |  | **11** | Rationale for choosing the reference standard (if alternatives exist) | Paper |
|  |  | **12a** | Definition of and rationale for test positivity cut-offs or result categories  of the index test, distinguishing pre-specified from exploratory | Paper |
|  |  | **12b** | Definition of and rationale for test positivity cut-offs or result categories  of the reference standard, distinguishing pre-specified from exploratory | Paper |
|  |  | **13a** | Whether clinical information and reference standard results were available  to the performers/readers of the index test | Paper |
|  |  | **13b** | Whether clinical information and index test results were available  to the assessors of the reference standard | Paper |
|  | *Analysis* | **14** | Methods for estimating or comparing measures of diagnostic accuracy | Paper |
|  |  | **15** | How indeterminate index test or reference standard results were handled | Paper |
|  |  | **16** | How missing data on the index test and reference standard were handled | Paper |
|  |  | **17** | Any analyses of variability in diagnostic accuracy, distinguishing pre-specified from exploratory | Paper |
|  |  | **18** | Intended sample size and how it was determined | Paper and Appendix |
|  | **RESULTS** |  |  |  |
|  | *Participants* | **19** | Flow of participants, using a diagram | Paper |
|  |  | **20** | Baseline demographic and clinical characteristics of participants | Paper |
|  |  | **21a** | Distribution of severity of disease in those with the target condition | Paper |
|  |  | **21b** | Distribution ofalternative diagnoses in those without the target condition | Paper |
|  |  | **22** | Time interval and any clinical interventions between index test and reference standard | Paper |
|  | *Test results* | **23** | Cross tabulation of the index test results (or their distribution)  by the results of the reference standard | Paper |
|  |  | **24** | Estimates of diagnostic accuracy and their precision (such as 95% confidence intervals) | Paper |
|  |  | **25** | Any adverse events from performing the index test or the reference standard | Paper |
|  | **DISCUSSION** |  |  |  |
|  |  | **26** | Study limitations, including sources of potential bias, statistical uncertainty, and generalisability | Paper |
|  |  | **27** | Implications for practice, including the intended use and clinical role of the index test | Paper |
|  | **OTHER INFORMATION** |  |  |  |
|  |  | **28** | Registration number and name of registry | Paper |
|  |  | **29** | Where the full study protocol can be accessed | Paper |
|  |  | **30** | Sources of funding and other support; role of funders | Paper |
|  |  |  |  |  |

## Methods

### Participants (8/9)

The project team was made aware of all admissions to neonatal units in the networks through the SEND database which was updated daily. It was interrogated daily by the Programme Manager and the neonatal units contacted to arrange a discussion with the parents, planned after the second week to avoid the difficult period around birth. This flexibility allowed parents to be approached at a time suitable for them and optimize their interaction with the research team.

A research nurse visited the referring unit and explained the study, including: randomization during the imaging visit; that information would be sought on perceptions and healthcare usage at term, one year, and at the visit for assessment at 20 months corrected age. This nurse was the principal contact with the family throughout the study, building a relationship essential for high follow-up rates.

The referral center was the Neonatal Imaging facility at Queen Charlotte’s and Chelsea Hospital. There were appropriate on-site patient and counseling rooms, and complete neonatal resuscitation facilities. Neurodevelopmental assessments were carried out at a number of sites including the Children’s Outpatient Department at Queen Charlotte’s and Chelsea Hospital, the Clinical Research Facility at St George’s Hospital, and in some cases the families’ homes.

### Sample size (18)

The original power calculation for this project was based on the following prior estimates: an incidence of cerebral palsy of 9% (based on data from the Epipage study^15^); sensitivity and specificity for predicting adverse motor outcome: 65% and 84%, and for moderate/severe cognitive deficits: (after Woodward^16^). Using these parameters, in a study of about 820 patients, MR has an estimated sensitivity with 95% CI, 54% to 75%, and specificity with 95% CI 81% to 87%. When the study sample size was revised after review of the first 234 patients and repeated measures ANCOVA chosen as an analysis method for the main trial the precision of diagnosis in the nested study was reviewed: 95% CI for sensitivity were predicted to be 52% to 77%; and specificity, 80% to 88%.

# CHEERS checklist and supplementary information

## CHEERS checklist

**CHEERS Checklist**

**Items to include when reporting economic evaluations of health interventions**

The **ISPOR CHEERS Task Force Report**, *Consolidated Health Economic Evaluation ReportingStandards (CHEERS)—Explanation and Elaboration: A Report of the ISPOR Health Economic Evaluations Publication Guidelines Good Reporting Practices Task Force,* provides examples and further discussion ofthe 24- item CHEERS Checklist and the CHEERS Statement. It may be accessed via the *Value in Health* or via the ISPOR Health Economic Evaluation Publication Guidelines – CHEERS: Good Reporting Practices webpage: <http://www.ispor.org/TaskForces/EconomicPubGuidelines.asp>

| **Section/item** | **Item no** | **Recommendation** | **Reported on** |
| --- | --- | --- | --- |
| **Title and abstract** |  |  |  |
| Title | 1 | Identify the study as an economic evaluation or use more specific terms such as “cost-effectiveness analysis”, and describe the interventions compared. | Not applicable as study not an economic evaluation |
| Abstract | 2 | Provide a structured summary of objectives, perspective, setting, methods (including study design and inputs), results (including base case and uncertainty analyses), and conclusions. | Paper/Appendix |
| **Introduction** |  |  |  |
| Background and objectives | 3 | Provide an explicit statement of the broader context for the study. | Paper |
|  |  | Present the study question and its relevance for health policy or practice decisions. | Paper |
| **Methods** |  |  |  |
| Target population and subgroups | 4 | Describe characteristics of the base case population and subgroups analysed, including why they were chosen. | Paper |
| Setting and location | 5 | State relevant aspects of the system(s) in which the decision(s) need(s) to be made. | Paper |
| Study perspective | 6 | Describe the perspective of the study and relate this to the costs being evaluated. | Appendix |
| Comparators | 7 | Describe the interventions or strategies being compared and state why they were chosen. | Appendix |
| Time horizon | 8 | State the time horizon(s) over which costs and consequences are being evaluated and say why appropriate. | Appendix |
| Discount rate | 9 | Report the choice of discount rate(s) used for costs and outcomes and say why appropriate. | Appendix |
| Choice of health outcomes | 10 | Describe what outcomes were used as the measure(s) of benefit in the evaluation and their relevance for the type of analysis performed. | Appendix |
| Measurement of effectiveness | 11a | *Single study-based estimates:* Describe fully the design features of the single effectiveness study and why the single study was a sufficient source of clinical effectiveness data. | Not applicable |
|  | 11b | *Synthesis-based estimates:* Describe fully the methods used for identification of included studies and synthesis of clinical effectiveness data. | Not applicable |
| Measurement and valuation of preference based outcomes | 12 | If applicable, describe the population and methods used to elicit preferences for outcomes. | Appendix |
| Estimating resources and costs | 13a | *Single study-based economic evaluation:* Describe approaches used to estimate resource use associated with the alternative interventions. Describe primary or secondary research methods for valuing each resource item in terms of its unit cost. Describe any adjustments made to approximate to opportunity costs. | Appendix |
|  | 13b | *Model-based economic evaluation:* Describe approaches and data sources used to estimate resource use associated with model health states. Describe primary or secondary research methods for valuing each resource item in terms of its unit cost. Describe any adjustments made to approximate to opportunity costs. | Not applicable |
| Currency, price date, and conversion | 14 | Report the dates of the estimated resource quantities and unit costs. Describe methods for adjusting estimated unit costs to the year of reported costs if necessary. Describe methods for converting costs into a common currency base and the exchange rate. | Appendix |
| Choice of model | 15 | Describe and give reasons for the specific type of decision-analytical model used. Providing a figure to show model structure is strongly recommended. | Not applicable |
| Assumptions | 16 | Describe all structural or other assumptions underpinning the decision-analytical model. | Not applicable |
| Analytical methods | 17 | Describe all analytical methods supporting the evaluation. This could include methods for dealing with skewed, missing, or censored data; extrapolation methods; methods for pooling data; approaches to validate or make adjustments (such as half cycle corrections) to a model; and methods for handling population heterogeneity and uncertainty. | Appendix |
| **Results** |  |  |  |
| Study parameters | 18 | Report the values, ranges, references, and, if used, probability distributions for all parameters. Report reasons or sources for distributions used to represent uncertainty where appropriate. Providing a table to show the input values is strongly recommended. | Not applicable |
| Incremental costs and outcomes | 19 | For each intervention, report mean values for the main categories of estimated costs and outcomes of interest, as well as mean differences between the comparator groups. If applicable, report incremental cost-effectiveness ratios. | Appendix |
| Characterising uncertainty | 20a | *Single study-based economic evaluation:* Describe the effects of sampling uncertainty for the estimated incremental cost and incremental effectiveness parameters, together with the impact of methodological assumptions (such as discount rate, study perspective). | Appendix |
|  | 20b | *Model-based economic evaluation:* Describe the effects on the results of uncertainty for all input parameters, and uncertainty related to the structure of the model and assumptions. | Not applicable |
| Characterising heterogeneity | 21 | If applicable, report differences in costs, outcomes, or cost-effectiveness that can be explained by variations between subgroups of patients with different baseline characteristics or other observed variability in effects that are not reducible by more information. | Appendix |
| **Discussion** |  |  |  |
| Study findings, limitations, generalisability, and current knowledge | 22 | Summarise key study findings and describe how they support the conclusions reached. Discuss limitations and the generalisability of the findings and how the findings fit with current knowledge. | Paper |
| **Other** |  |  |  |
| Source of funding | 23 | Describe how the study was funded and the role of the funder in the identification, design, conduct, and reporting of the analysis. Describe other non-monetary sources of support. | Appendix (Consort) |
| Conflicts of interest | 24 | Describe any potential for conflict of interest of study contributors in accordance with journal policy. In the absence of a journal policy, we recommend authors comply with International Committee of Medical Journal Editors recommendations. | Not applicable at this stage |
|  |  |  |  |

**Note: Our study is not a full economic evaluation and it describes health care resource, costs and quality of life and costs associated to providing or not MRI information to mothers of preterm babies. However, most of the sections of the CHEERS statement apply and is included for the benefit of the editor and reviewers.**

# Governance Committees

## Trial Steering Committee

Prof Richard Cooke (Chair), David Edwards, Denis Azzopardi, Nigel Kennea, Nuria Gonzalez-Cinca

## Data monitoring and Ethics Committee

Prof A George (Chair), Prof D Walters, Dr R Hurlston.

# Reference list

1. Drummond MF, Sculpher M, Claxton K, Stoddart GL, Torrance GW. Methods for the economic evaluation of health care programmes. Oxford: Oxford University Press; 2015.

2. Imperial College Healthcare NHS Trust. Women’s and Children’s Division. Children’s Services. London: Imperial College Healthcare NHS Trust; 2013.

3. Curtis L. Unit Costs of Health and Social Care. University of Kent: Personal Social Services Research Unit; 2013.

4. NHS Reference costs 2013-14. 2015. at <https://www.gov.uk/government/publications/nhs-reference-costs-2013-to-2014>.)

5. National Institute for Health and Care Excellence. Guide to the methods of technology appraisal. London: National Institute for Health and Care Excellence; 2013.

6. Husereau D, Drummond M, Petrou S, et al. Consolidated Health Economic Evaluation Reporting Standards (CHEERS)--explanation and elaboration: a report of the ISPOR Health Economic Evaluation Publication Guidelines Good Reporting Practices Task Force. Value Health 2013;16:231-50.

7. Brooks R. EuroQol: the current state of play. Health Policy 1996;37:53-72.

8. Dolan P. Modeling valuations for EuroQol health states. Medical Care 1997;35:1095-108.

9. Jenkinson C, Layte R. Development and testing of the UK SF-12. JHealth ServResPolicy 1997;2:14-8.

10. Brazier JE, Roberts J. The Estimation of a Preference-Based Measure of Health from the SF-12. Medical Care 2004;42:851-9.

11. Nixon RM, Wonderling D, Grieve RD. Non-parametric methods for cost-effectiveness analysis: the central limit theorem and the bootstrap compared. Health Econ 2010;19:316-33.

12. White IR, Royston P, Wood AM. Multiple imputation using chained equations: Issues and guidance for practice. Stat Med 2011;30:377-99.

13. Faria R, Gomes M, Epstein D, White IR. A guide to handling missing data in cost-effectiveness analysis conducted within randomised controlled trials. Pharmacoeconomics 2014;32:1157-70.

14. Little RJ, Rubin DB. Statistical Analysis with Missing Data. 2nd ed. Hoboken, NJ: Wiley; 2002.

15. Larroque B, Ancel PY, Marret S, et al. Neurodevelopmental disabilities and special care of 5-year-old children born before 33 weeks of gestation (the EPIPAGE study): a longitudinal cohort study. Lancet 2008;371:813-20.

16. Woodward LJ, Anderson PJ, Austin NC, Howard K, Inder TE. Neonatal MRI to predict neurodevelopmental outcomes in preterm infants. N Engl J Med 2006;355:685-94.

# SUPPLEMENTARY TABLES

Table S1: Parameters for MR imaging

| **Sequence** | **TR**  **(ms)** | **TE**  **(ms)** | **Flip angle** | **Number of slices** | **Slice Thickness (mm)** | **FOV**  **(mm)** | **Matrix** | **Voxel size** |
| --- | --- | --- | --- | --- | --- | --- | --- | --- |
| T2 weighted fast spin echo | 14730 | 160 | 90 | 92 | 2 | 220x220 | 256x256 | 0.86/0.86/2 |
| T1 high resolution volume(3D MPRAGE) | 17 | 4.6 | 13 | 240 | 0.8 | 210x210 | 256x256 | 0.82/0.82/0.8 |
| Dynamic single shot fast spin echo | shortest | 160 | 90 | 92 | 2 | 220x210 | 256x256 | 0.86/0.86/2 |

T1 and T2: signal weighting; TR (Repetition time) and TE (Echo time): sequences parameters; FOV: field of view; mm: millimeters; ms: milliseconds

Table S2: Summary of categories of health care resource use items and unit costs used in the cost-analysis (expressed in 2013/2014 UK prices)

| **Resource use item** | **Unit cost** | **Sources** | **Notes** |
| --- | --- | --- | --- |
| **Community professional care** |  |  |  |
| General practitioner | £67.00 | 1 | Per typical 17.2 min. consultation |
| Health visitor | £50.00 | 1 | Per hour/contact |
| Practice nurse | £41.00 | 1 | Per hour/contact |
| Community nurse | £50.00 | 1 | Per hour/contact |
| Home visitor/volunteer | £30.00 | 1 | Per hour/contact (note: ‘family support worker’) |
| Community paediatrician | £288.61 | 2 | Per consultant contact |
| Physiotherapist | £37.00 | 1 | Per hour/contact (note: ‘hospital physiotherapist’) |
| Social worker | £57.00 | 1 | Per hour/contact (note: social worker for children’s services) |
| Other visits | £77.57 | 1 | Average of community care visits |
| **Secondary care** |  |  |  |
| Accident and Emergency | £111.91 | 2 | Weighted average of unit cost of all non-admitted to emergency medicine |
| Routine hospital follow-up | £175.09 | 2 | Paediatric Non-Admitted Face to Face Attendance, Follow-up |
| Hospital outpatient | £235.25 | 2 | Paediatric Non-Admitted Face to Face Attendance, First |
| Hospital in-patient stay (per day) | £2,708 | 2 | Weighted average of unit cost of all non-elective long stay paediatric inpatient admissions |
| Hospital in-patient operations (per day) | £2,595 | 2 | Weighted average of unit cost of all elective paediatric inpatient admissions |

Sources:

[1] Curtis L, (2014). Unit Costs of Health and Social Care. University of Kent, Personal Social Services Research Unit.

[2] Department of Health (2014). "NHS Reference costs 2013."https://www.gov.uk/government/publications/nhs-reference-costs-2013-to-2014”.

Table S3: Maternal health-related quality of life using EQ-5D-3L and SF-6D scores at 12 and 20 months follow-up using multiple imputation analysis

| **Quality of life instrument** | **MRI (n=255)**† | | | **Ultrasound (n=255)** † | | | **Mean difference**  **(95% CI)** |
| --- | --- | --- | --- | --- | --- | --- | --- |
|  | n | Mean | SE | n | Mean | SE |  |
| EQ-5D-3L score 12 months | 255 | 0.902 | 0.013 | 255 | 0.919 | 0.111 | -0.016 (-0.050 to 0.017) |
| EQ-5D-3L score 20 months | 255 | 0.922 | 0.011 | 255 | 0.922 | 0.010 | 0.000 (-0.030 to 0.030) |
| SF-6D score 12 months | 255 | 0.786 | 0.010 | 255 | 0.779 | 0.009 | 0.007 (-0.021 to 0.035) |
| SF-6D score 20 months | 255 | 0.793 | 0.008 | 255 | 0.770 | 0.008 | 0.022 (-0.001 to 0.079) |

MRI: Magnetic resonance imaging; SE: Standard Error; CI: parametric confidence interval; †: number of infants in each group (MRI and ultrasound).

Table S4: Breakdown of infant healthcare resource use consumption over the past 24 months since leaving neonatal care, complete cases only

|  | **MRI (n=255) ^†^** | | | | |  | **Ultrasound (n=255) ^†^** | | | | |  |
| --- | --- | --- | --- | --- | --- | --- | --- | --- | --- | --- | --- | --- |
|  | n | Missing | Min | Max | Mean (SD) |  | n | Missing | Min | Max | Mean (SD) | pvalue  (mean diff) |
| **Community professional** |  |  |  |  |  |  |  |  |  |  |  |  |
| General Practice | 142 | 113 | 0 | 95 | 9.33 (12.07) |  | 162 | 93 | 0 | 55 | 8.41 (7.19) | 0.41 |
| Health visitor | 138 | 117 | 0 | 52 | 7.15 (6.75) |  | 155 | 100 | 0 | 36 | 6.97 (5.94) | 0.81 |
| Practice nurse | 132 | 123 | 0 | 14 | 2.49 (2.44) |  | 139 | 116 | 0 | 21 | 2.73 (2.73) | 0.44 |
| Community nurse | 125 | 130 | 0 | 48 | 1.70 (5.64) |  | 134 | 121 | 0 | 20 | 1.66 (3.74) | 0.94 |
| Home visitor/volunteer | 123 | 132 | 0 | 88 | 1.05 (8.00) |  | 134 | 121 | 0 | 48 | 1.13 (5.75) | 0.92 |
| Community paediatrician | 129 | 126 | 0 | 8 | 1.40 (2.21) |  | 136 | 119 | 0 | 21 | 1.09 (2.55) | 0.33 |
| Physiotherapist | 131 | 124 | 0 | 39 | 2.97 (6.15) |  | 145 | 110 | 0 | 82 | 3.48 (10.71) | 0.63 |
| Social worker | 127 | 128 | 0 | 9 | 0.28 (1.40) |  | 139 | 116 | 0 | 4 | 0.10 (0.57) | 0.18 |
| Other visits | 125 | 130 | 0 | 25 | 1.73 (4.40) |  | 140 | 115 | 0 | 47 | 2.04 (5.81) | 0.62 |
| **Secondary care** |  |  |  |  |  |  |  |  |  |  |  |  |
| Routine hospital follow-up visit | 151 | 104 | 0 | 40 | 5.21 (5.63) |  | 160 | 95 | 0 | 32 | 4.79 (4.03) | 0.45 |
| Accident and Emergency | 131 | 124 | 0 | 32 | 2.76 (4.52) |  | 153 | 102 | 0 | 21 | 2.41 (2.81) | 0.43 |
| Other hospital outpatient visit | 141 | 114 | 0 | 18 | 1.54 (2.95) |  | 149 | 106 | 0 | 21 | 1.58 (2.95) | 0.90 |
| Hospital in-patient admission (in days) | 168 | 87 | 0 | 82 | 3.41 (8.77) |  | 185 | 70 | 0 | 31 | 2.35 (5.13) | 0.16 |
| Operations length of stay (in days) | 168 | 87 | 0 | 45 | 0.57 (3.58) |  | 181 | 74 | 0 | 11 | 0.45 (1.47) | 0.70 |
| **Prescriptions** |  |  |  |  |  |  |  |  |  |  |  |  |
| Frequency of prescribed medication | 190 | 65 | 0 | 8 | 1.26 (1.63) |  | 199 | 56 | 0 | 8 | 1.50 (1.82) | 0.17 |
| Number of days using medication | 187 | 68 | 0 | 1994 | 148.93 (310.35) |  | 197 | 58 | 0 | 1646 | 175.97 (330.48) | 0.41 |

MRI: Magnetic resonance imaging; SD: Standard Deviation; †: number of infants in each group (MRI and ultrasound).

Table S5: Cost-analysis over the past 20 months since leaving neonatal care expressed in 2013/2014 UK prices using multiple imputation analysis

|  | **MRI (n=255) ^†^** |  | **Ultrasound (n=255) ^†^** |  |  |
| --- | --- | --- | --- | --- | --- |
|  | Mean (SE) |  | Mean (SE) | Mean difference | 95% CIs |
| **Cost of intervention** | £773 (£0) |  | £458 (£11) | £315 | (£295 to £336) |
| **Community professional** |  |  |  |  |  |
| General Practice | £702 (£82) |  | £553 (£35) | £150 | (-£28 to £327) |
| Health visitor | £358 (£26) |  | £338 (£22) | £20 | (-£48 to £87) |
| Practice nurse | £104 (£9) |  | £109 (£8) | -£5 | (-£28 to £19) |
| Community nurse | £85 (£24) |  | £87 (£16) | -£2 | (-£57 to £53) |
| Home visitor/volunteer | £40 (£20) |  | £44 (£15) | -£4 | (-£53 to £45) |
| Community paediatrician | £383 (£55) |  | £332 (£59) | £50 | (-£111 to £211) |
| Physiotherapist | £125 (£20) |  | £113 (£27) | £12 | (-£56 to £79) |
| Social worker | £18 (£7) |  | £5 (£2) | £12 | (-£2 to £27) |
| Other visits | £153 (£31) |  | £176 (£38) | -£22 | (-£116 to £71) |
| **Secondary care** |  |  |  |  |  |
| Routine hospital follow-up visit | £883 (£70) |  | £807 (£49) | £77 | (-£92 to £246) |
| Accident and Emergency | £310 (£57) |  | £262 (£23) | £48 | (-£76 to £172) |
| Other hospital outpatient visit | £394 (£67) |  | £358 (£54) | £36 | (-£137 to £210) |
| Hospital in-patient admission | £9,399 (£1,814) |  | £6,140 (£966) | £3,259 | (-£607 to £7,125) |
| Operations length of stay | £2,506 (£1,057) |  | £1,136 (£276) | £1,369 | (-£781 to £3,520) |
| **Total cost per preterm baby** | £16,231 (£2,595) |  | £10,916 (£1,239) | £5,315 | (-£188 to £10,819) |

MRI: Magnetic resonance imaging; SE: Standard Error; CI: parametric confidence interval; †: number of infants in each group (MRI and ultrasound).

Table S6: Maternal confidence and perceptions of singleton infants and their care (Questionnaire data from 4 time points)

| **Confidence and perceptions** | **MRI**  **(n=178)** ^††^ | | **Ultrasound**  **(n=188)**^††^ |
| --- | --- | --- | --- |
| **How confident did you feel about caring for your baby in the neonatal unit; n (%)- pre-imaging** | | | |
| Very confident | 136 (94.4) | | 146 (90.7) |
| Quite confident | 8 (5.6) | | 14 (8.7) |
| Not confident | 0 | | 1 (0.6) |
| Missing, n: | 34 | | 27 |
| **How confident do you feel about these aspects of caring for your baby now? Mean [SD] 14 days after imaging** (6 items scored 0-4)   \| Total score  Missing, n: \| 16.66 [2.22]  28 \| 16.63 [2.41]  22 \| \| --- \| --- \| --- \|   **How would you describe your baby at the moment?; Mean [SD] 14 days after imaging** (24 items scored 1/0) | | | |
| Positive words selected | 63.49 [25.23] | | 60.25 [26.33] |
| Negative words selected | 15.63 [17.57] | | 16.17 [18.47] |
| Missing, n: | 26 | | 21 |
| **How confident do you feel about these aspects of caring for your baby now? Mean [SD] 12 months after imaging**  (7 items scored 0-4) | | | |
| Total score  Missing, n: | 17.32 [1.86]  36 | 17.06 [1.97]  27 | |
| **How would you describe your baby at the moment?; Mean [SD] 12 months after imaging** (24 items scored 1/0) | | | |
| Percentage of positive words selected | 67.02 [18.10] | | 65.37 [19.42] |
| Percentage of negative words selected | 9.67 [8.01] | | 7.76 [7.40] |
| Missing, n: | 35 | | 27 |
| **How confident do you feel about these aspects of caring for your baby now? Mean [SD] 20 months after imaging** (5 items scored 0-4) | | | |
| Total score  Missing, n: | 14.37 [1.23]  11 | | 14.23 [1.51]  5 |
| **How would you describe your baby at the moment? Mean [SD] 20 months after imaging** (24 items scored 1/0) | | | |
| Percentage of positive words selected | 65.47 [17.95] | | 64.30 [19.45] |
| Percentage of negative words selected | 12.23 [9.58] | | 10.20 [10.18] |
| Missing, n: | 11 | | 5 |

SD: standard deviation; ††: number of mothers with primary outcome data (STAI) in each group at baseline, 14 days, 12 months and 20 months corrected aged.
